# Impact of Interpersonal Competition on Knowledge Hiding Behavior Among the Employees: Mediating Role of Moral Disengagement and Work Overload

**DOI:** 10.3389/fpsyg.2022.881220

**Published:** 2022-04-27

**Authors:** YiFan Wang

**Affiliations:** School of Humanities, Southeast University, Nanjing, China

**Keywords:** interpersonal competition, knowledge hiding behavior, moral disengagement, work overload, employee performance

## Abstract

The knowledge hiding behavior (KHB) can obstruct the stream of information to decrease the creativity in the organization. This study examines the effect of interpersonal competition on KHB, moral disengagement (MD), and work overload (WO). Moreover, this study also examines the impact of MD and WO on KHB. Also, the study examines the mediating role of MD and WO between interpersonal competition and KHB. The study was carried out by quantitative methodology, and 361 employees were engaged to fill the questionnaires employed in manufacturing companies from China. A convenient sampling technique had used for data collection. The findings of this study indicate that interpersonal competition positively and significantly affects KHB, MD, and WO. Moreover, this study established that MD and WO positively and significantly impact KHB. According to the results, MD WO significantly mediates interpersonal competition and KHB. This research is valuable for government, policymakers, and executives of manufacturing companies to establish the appropriate strategies for employees and provide a sustainable environment. This research also offers new visions to managers to know the current events and predict the possible causes that lead to the KHB and what is the possible strategies to eliminate this kind of behavior.

## Introduction

In today’s world of fierce competition, the progressing globalization and intense technological advancement have made knowledge a vital tool, driving the firms’ operations. To the developing significance of knowledge in numerous disciplines, various organizations have contributed to knowledge creation to achieve business success. Knowledge, a valuable resource, fosters the theoretical understanding of the subject, thereby upgrading an individual’s behavior and status (Raza et al., 2018). Undoubtedly, knowledge being a worthwhile asset is an impulsion that makes employees compete successfully in the business environment.

However, in the recent years, internal competition has spurred the employees in power, with knowledge assets working as the incentive mechanism, accelerating the enterprise activities. In particular, competition is an activity that establishes supremacy and rivalry (e.g., internal and external) over the others. Accordingly, interpersonal competition alludes to the rivalry between the two parties attempting to acquire superiority at the workplace. It motivates individuals to compete for resources, incentives, position, and power, thus accomplishing organizational goals. Perhaps, it is a powerful driver that leads individuals to see their co-workers as their immediate competition (Sarfraz et al., 2019; Shah et al., 2019; Kalra et al., 2021).

Indeed, contrary to knowledge sharing, today, the progressing internal competitiveness has fostered the concept of knowledge hiding, thus elevating the need for effective knowledge management. Knowledge hiding is a counterproductive behavior that encourages employees to limit knowledge sharing to gain supremacy (Singh, 2019). Knowledge largely depends on the individual intention to share the resource to gain organizational reward and benefit (Lanke, 2018). Considerably, knowledge hiding behavior (KHB) alludes to an act of intentionally withholding the knowledge as requested by a fellow member. Knowledge hiding is an accepted phenomenon that is widely prevalent in the work setting (Jiang et al., 2019; Ajaz et al., 2020). Given the articulation, the research states that employees face difficulty sharing important information with their subordinates, thus predicting increased interpersonal competitiveness (Connelly et al., 2019). Perhaps, it is a subtle way of rationalizing the individuals’ inability to share information among individuals, groups, and organizations (Anand and Hassan, 2019).

For the organization to gain competitiveness, the management expects employees to exchange knowledge with the other workers (Khalid et al., 2018). Consistently, KHB enables individuals to forgo their responsibility of helping others. This action leads to the concept of moral disengagement (MD), which alludes to the process that makes individuals bear the consequences of their immoral violations. It is a phenomenon that enables individuals to disengage from the process of morality, leading them to act against moral standards (Bandura et al., 1996). It makes individuals think that their actions are ethically justifiable (Sahi and Ahmad, 2019; Abdullah et al., 2021). The study shows that MD fosters immoral motives in individuals, thus making them exhibit behavior (i.e., unethical) which is inhumane and morally unacceptable.

Apart from growing MD, today, the developing competitiveness has also increased the workload on employees. Consistent with this statement, the research shows that inability to cope with the increasing job demands encourages individual to hide their knowledge from other employees (Jahanzeb et al., 2020). Work overload (WO) refers to the employees’ inability to deal with the work burden (Spector and Jex, 1998). It refers to long hours’ work, a waste of time, and a sense of frustration, thus adversely affecting employee productivity and cognition (Abdullah et al., 2018a; Hobfoll et al., 2018). Indeed, it fundamentally influences the firms’ working environment, thereby generating a need to adopt novel approaches to deal with this issue.

Research on knowledge management has considerably increased over the last few years. Numerous determinants had predicted different approaches involving knowledge-sharing behavior. Perhaps, despite the expanding literature on knowledge sharing, there is still an extended scope to explore in the context of KHB. The literature shows that limited literature has examined the effect of knowledge hiding in the competitive work setting (Han et al., 2020). Similarly, another study states that a small mechanism had illustrated this concept that suggests bringing the research into the limelight that speaks on the notion of knowledge hiding (Bavik, 2020). Today, the increased focus on knowledge in the competitive world requires to dig deep into the literature of knowledge management behavior (Hernaus et al., 2019). Indeed, our research reveals that very few studies have investigated the effect of knowledge hiding in today’s business environment (Boz Semerci, 2019). Accordingly, the previous studies call empirical literature to demonstrate the numerous aspects of KHB influencing various factors. Consequently, this study emphasizes a need for deep research on employees’ KHB (Shrivastava et al., 2021).

However, in response to filling the research gap, this study empirically aims to follow the instructions delivered to discover the KHB in the internal competitive environment. The study objective is to explore the different factors that lead to KHB among employees. As opposed to prior studies, the study objective was to examine the direct relationship of interpersonal competition with MD and WO. However, previous literature calls for a study that explores the mediating role of knowledge hiding in the competitive work setting. Besides the claims made in the literature, very few empirical studies have integrated the mediating effect of MD. This study extends the association between knowledge hiding and interpersonal competition by substantially investigating the mediating influence of MD. The theoretical framework fundamentally evaluates the mediating effect of WO, arching the nexus between interpersonal competition and KHB. Significantly, this study bridges the research gaps by presenting a well-built establishment on the construct of knowledge hiding, MD, and WO.

Subsequently, to the best of our knowledge, this study is a novel contribution toward knowledge management. Its research objective equipped with the enriched literature builds a strong knowledge foundation, making this study a unique presentation of the prior studies. This study presents a framework suggesting the best approach to investigate the research hole. Its extended scope broadens the knowledge on the proposed concept by accumulating the dispersed literature from the management domain. It fundamentally integrates the scattered data under one research topic. Following the necessary debate and need for theoretical contribution, this article is a well-written presentation of a novel conceptual model for the practitioners across the management discipline. It suggests that establishing a friendly knowledge-sharing culture requires practitioners to integrate high knowledge skills, thus ensuring the dynamic practice of moral engagement in complex situations.

Significantly, this unique model is a valuable addition to the existing literature that compels this knowledge to aid the organizations, lessening the deliberate effect of knowledge hiding. The study elements capture the attention of academicians and future researchers, thus improving their understanding of the maturing field of knowledge hiding in the competitive environment. Further, this article presents the impact of interpersonal competition on executives in a structural form. The research findings make them realize impeding factor that causes interpersonal competition to increase. Consequently, this study holds immense significance for individuals, groups, and organizations by providing a broader understanding of the knowledge culture in the workplace setting.

Hence, as a quick reminder, this study starts with introducing the study concept. Along with this, the second section (i.e., literature review) illustrates the theoretical background by explicitly developing a diverse set of hypotheses involving interpersonal competition. In the same vein, the study methodology (refer to section “Methodology”) describes the relevant research instruments needed for study analysis. Similarly, section “Results” demonstrates the research results, with section “Discussion” discussing the study outcomes. Finally, section “Conclusion and Implications” concludes the research paper by presenting implications for future practice.

## Literature Review

Knowledge is the core element that enables the firm to achieve sustainability in the growing business environment. Undoubtedly, the employees’ knowledge performance plays a crucial role in fostering the firms’ growth (Pérez-Salazar et al., 2019). Workers’ knowledge supports the corporations’ success. But, still, some employees hide knowledge for their interests and welfare. Consequently, section “Literature Review” formulates a strong research background, thereby establishing a direct and indirect relationship in the light of previous studies’ reviews. The following section provides a detailed overview of the intended terms: interpersonal competition (IC), KHB, MD, and WO. Indeed, to capture the readers’ attention, all the details had demonstrated in the same series in the below section.

### Interpersonal Competition and Knowledge Hiding Behavior

Over the years, the significance of knowledge sharing accelerating the firms’ activities has considerably gained management attention. Despite the various attempts to strengthen individual ability, knowledge sharing has led KHB, a new concept, to the limelight (Anand et al., 2020). Predominately, it refers to the intentional hiding of knowledge requested by the fellow member for achieving power, ownership, and success (Jahanzeb et al., 2020). Consistently, the study shows that employees hide knowledge due to insufficient rewards of sharing knowledge, the increasing rivalry, and psychological entitlement (Abdullah et al., 2018b; Wen and Ma, 2021).

Knowledge hiding is a novel construct influenced by the interpersonal environment. Employees refrain from sharing the knowledge to increase their work status, performance, and position. The interpersonal competition motivates the knowledge holder to hide the information to maintain ownership. Potentially, this action enhanced by internal competition implies that this negative attitude of hiding the knowledge reduces individuals’ power. The favored hiding of the knowledge drives the employees to feel obstructive and helpless. Given the statement, the research states interpersonal competition to be a positive stimulator of KHB (Hernaus et al., 2019). However, various factors trigger KHB. Out of all, interpersonal competition is the most significant factor driving the employees’ intention to obscure knowledge (Jha et al., 2019). Indeed, this undesirable action is a common phenomenon among the employees that creates a barrier to knowledge sharing behavior.

Perhaps, to thrive in the competitive economy, where interpersonal competition is at its peak, KHB presents a disincentive to the organization, thus hampering its performance and sustainability. In particular, the organization’s success is highly dependent on employees’ knowledge-sharing behavior. However, besides the numerous incentives of knowledge sharing, the literature shows that employees are still reluctant to share the information. In explaining this notion, the study states that the increased internal competition leads employees to hide the information, substantially making firms experience the diminished result of knowledge hiding (e.g., reduced productivity; Kumar Jha and Varkkey, 2018). Hence, a knowledge-based environment has become vital for the institution’s progress.

The KHB enables the individuals to limit the crucial information to themselves, thus gaining superiority over the other employees. In the illustration, the study shows that the increased workplace competition makes the individual hide the knowledge, potentially thinking the other person to be his competitor (Xiao and Cooke, 2019). Similarly, the research shows that a highly competitive environment (e.g., internal) makes the employees hide the knowledge at the workplace (Swab and Johnson, 2019). Arguably, competitive work knowledge leads individuals to achieve personal goals, thus pursuing competitive advantage. Perhaps, staying ahead of others leads the employees to engage in unacceptable behavior. KHB makes employees keep information to themselves, substantially withholding an edge over the other colleagues (Hernaus et al., 2019).

Considerably, the escalating interpersonal pressure encourages individuals to exhibit destructive behavior (Hernaus et al., 2019). Personal competitiveness makes individuals respond selfishly to the increasing requirement of the workplace. This act shows that the individual is too busy to exchange knowledge with colleagues. In the illustration, the study demonstrates that a competitive climate leads individuals to exhibit KHB (Xiong et al., 2021). Hence, the literature presents a strong background on KHB among the employees due to the accelerating internal competition. Indeed, based on the prior research, we had formulated the following hypothesis.

*H1: Interpersonal competition has a positive and significant impact on knowledge hiding behavior.*

### Interpersonal Competition and Moral Disengagement

Remarkably, in the past few years, considerable research has accumulated on the increasing significance of interpersonal competition for understanding the role of MD. At Present, ethics scholars have drawn attention to the growing prominence of interpersonal competition leading to MD behaviors. MD refers to the individual behavior violating ethical standards. In the firms’ competitive environment, ethics makes the individuals sustain a superior position. The unethical tactics lead the individuals to provide false information, thus giving rise to ethical business dilemmas. IC drives unethical distortions to motivate employees to exhibit unethical behavior (Li et al., 2018). Given the statement, the study states that employees’ competitive orientation significantly amplifies the MD act (Liu and Kim, 2020).

Furthermore, within the organization, moral cognition significantly influences employees’ behavior. Consistently, a rapid increase in morality awareness ensures the implementation of ethical conduct at the workplace. The employee perception of increased competition influences their ethical consideration. Employees facing intense personal rivalry foster unethical choices, thus deviating from the ethical standards. Given the statement, the research states that antisocial behavior elevates the negative attitude, thus evoking MD in individuals (Shehzad et al., 2020; van de pol et al., 2020). Likewise, interpersonal rivalry makes the individual secure their interest while prompting unethical behavior at the workplace. Given the explanation, the study shows that individual self-interest makes the employees activate MD, thus engaging in immoral practices (Khan et al., 2021). In a competitive environment, the employees’ unethical behavior motivates them to act immorally, thus ensuring personal motives. This detrimental behavior restricts the individual from performing the activities as per the standardized rules, thus leading individuals to be inconsistent with moral standards (Scheiner et al., 2018).

In particular, MD, being widespread, has a considerable impact on organizations and stakeholders (i.e., employees). At the individual level, engaging in illegal activities is the function of interpersonal competition. Competition elevates the feeling of frustration in individuals. Employees feeling competitive pressure reciprocate their behaviors against moral principles. Indeed, the competitive orientation demonstrates employees’ desire to maximize personal interest, thus ignoring the ethical standards. Therefore, the hypothesis developed based on the prior studies reviews concludes.

*H2: Interpersonal competition has a positive and significant impact on moral disengagement.*

### Interpersonal Competition and Work Overload

Interpersonal competition is the most studied topic across the various management discipline. Accordingly, many scholars have developed considerable literature on the firms’ competitive environment. As per the prior literature, the consequences of interpersonal competition are appraised as a challenge to downsize the effects of organizational rewards. WO increases the interpersonal tension among individuals. Given the explanation, the study shows that interpersonal competition makes the individuals exhibit hostile behavior (e.g., disagreements, conflicts) toward co-workers (Wallace and Buchanan, 2019). Hence, it is a controversial force creating rivalry among the workers.

However, WO has become employees’ prime concern in the recent years that demands management attention. Heavy workloads require individuals to exert extra time and effort, thus providing them fewer opportunities to excel in their job roles. The progressing interpersonal competition has overburdened the employees, thus making it difficult for them to cope with the high workplace competitiveness. WO makes individuals feel unsatisfied with the work. Perhaps, in the review of the current literature, the study indicates that a competitive environment makes employees experience excessive workload and job stress (Qasim et al., 2020), thus making it hard for employees to meet the organizations’ standards.

The accelerating workplace subjects the individual to deal with the increasing work pressure such as intense competition. Competitive pressure amplifies the work strain, thus bringing adverse outcomes. Altogether, workload and competition initiate feelings of stress among the individuals. Given the statement, the research shows that internal competitiveness leads the individual to work under a competitive climate, subsequently fostering the feeling of anxiety and stress among the workforce (Babalola et al., 2022). In the changing business dynamics, the increasing workloads and long working hours have posed considerable pressure on employees, thus making them bear the work-related strain (Converso et al., 2018). Hence, to explain this notion, the study shows that individual job demand increases in the internal competitive climate, thus making it difficult for the employees to bear the excessive workload (Bunjak et al., 2021). Therefore, based on this, the research findings suggest.

*H3: Interpersonal competition has a positive and significant impact on work overload.*

### Moral Disengagement and Knowledge Hiding Behavior

Moral disengagement is a phenomenon that elevates the harmful effect of unethical attitudes. The MD model enables the employees to violate the ethical standards, thus leading it as an efficient predictor of deviant behavior (e.g., unethical, aggressive). Extant literature demonstrates the relation of MD with employee KHB. It states that the MD mechanism potentially records an increase in unethical doings (i.e., knowledge hiding). It makes employees free from feeling the guilt of unaccountability (Harris and He, 2019). Given the illustration, the study shows that unethically disengagement makes the individuals exhibit unethical behavior (e.g., KHB) without the feeling of sorrow (Qin et al., 2020).

Undoubtedly, knowledge hiding is the consequence of negative behavior. Knowledge is a profound consideration that influences individual behavior and activities (Raza et al., 2018). Given the articulation, the study indicates the wide range of unethical behavior predicated by MD elevates the KHB (Arain et al., 2020). Moreover, employee knowledge capability generates benefits for the organizations. In particular, the emerging benefits require the employees to ensure the proper implementation of ethical standards. Significantly, moral actions enable the organization to excel in today’s competitive environment. Violation of the norms in the competitive market affects the corporation’s position and employees’ goals (Khan et al., 2018). Indeed, the literature makes it vital for the management to understand the counterproductive behavior (i.e., knowledge hiding), leading an individual to exhibit an unethical code of conduct. However, an effective strategy needs to develop in supporting and handling unethical behavior in organizations. An ethical code of conduct curbs the employees’ unethical behavior, thus enhancing the firm market position. Therefore, managing moral practices have become critical for organizations’ success.

Furthermore, MD vigorously affects the employee’s capability of knowledge generation. Given the statement, the researchers suggest that reducing the positive ethical attitude manifests the employees’ KHB (Burmeister et al., 2018). Accordingly, the study suggests that the increased MD requires a self-regulatory mechanism for monitoring the employees’ moral actions (Moore et al., 2019). In particular, ethically disengaged employees avoid following ethical business practices, thus exhibiting unacceptable behavior. In the illustration, prior studies reveal a positive impact of MD with KHB (Jabeen and Anwar-ul-Haq, 2021). Therefore, from the above literature, we draw our conclusion as follows.

*H4: Moral disengagement has a positive and significant impact on knowledge hiding behavior.*

#### The Mediating Role of Moral Disengagement

In the era of fierce competition, the organization’s success considerably depends on the firm’s knowledge resources. Efficient knowledge ensures the organization’s growth and competitiveness. However, in an internally competitive environment, the knowledge holders refrain from sharing work-related information with their co-workers. The act of personal incompatibility raises the phenomenon of MD. The study shows that when employees indulge in competition, they tend to exhibit MD, thus making them hide knowledge (Li et al., 2018). In particular, MD leads to KHB. The study indicates that MD promotes unethical behavior (i.e., knowledge hiding) without making an individual feel guilty about his act (Zhao and Xia, 2019).

Considerably, immoral action makes the individual indulge in unethical activities, thus bringing detrimental outcomes. The moral agreement makes the individual act according to moral principles. However, people do not always act as to moral standards. In an unethical competitive environment, employees exhibit counterproductive behavior without morally following the ethical principles. Following this argument, the study reveals that the internal competitive environment elevates MD, fostering unethical behavior among the workers, thereby bringing adverse outcomes (Arain et al., 2020). Therefore, interpersonal competition leads the individual to be detached from ethical conduct, thereby facilitating the KHB.

In the competitive business setting, employees hiding knowledge poses an intense threat to personal and social welfare. Such action of hiding the information hinders the firms’ performance, thus impeding its competitiveness (Connelly et al., 2019). Individuals who undervalue the organizational reward express MD. Employees may unethically hide the knowledge if they do not perceive the firms’ returns as beneficial. Given the articulation, the study shows that this organizational injustice makes the employees hide the information (Rani et al., 2018), thus leading this notion to increase MD. In particular, MD encourages negative behavior and ethical misconduct, thus decreasing knowledge sharing in the work setting. Hence, based on the previous literature, the hypothesis suggests.

*H4a: Moral disengagement mediates the relationship between interpersonal competition and knowledge hiding behavior.*

### Work Overload and Knowledge Hiding Behavior

The developing technological advancement has made individuals bear the burden of workplace duties. The lack of work recognition makes the individual invest less effort in work, thus hampering their work performance. Today, the increasing job demands have compelled individuals to avoid performing extra-role duties such as helping others. In explaining this notion, the study shows that excessive WO contributes to KHB, thus limiting the individual to exert extra effort (Kumar Jha and Varkkey, 2018).

The excessive workload leads an individual to hide the workplace knowledge, thus depleting organizations’ productivity. Management expects workers to share the knowledge for accelerating the organization’s growth. Employees holding the organization’s information negatively influences the performance targets. The most crucial factor for gaining productivity is time resources. The WO puts the individual’s resources at risk. Knowledge sharing reduces the individual’s time for performing the task. Contrastingly, the excessive workload alludes to the belief that performing a task demands time and energy (Engelbrecht et al., 2020). This belief convinces the employees to experience increasing time constraints and energy loss. This work burden makes employees invest extra time (i.e., long working hours), thus fulfilling the work-related duties. In particular, employees’ unwillingness to put in extra hard work encourages them to hide organizational knowledge.

Furthermore, stress is a psychological factor relating to the workload. It directs the individual behavior for dealing with work-related burdens. The workload pressure elevates the workplace stressors, thus encouraging KHB. The employee’s reaction to excessive workload arises psychological challenges for employees. Given the statement, the study shows that employee job demands elevate the stress in individuals, thus leading them to limit sharing the knowledge from their co-workers (Feng and Wang, 2019). Hence, the WO leads individuals to experience excessive exhaustion, thus withdrawing from sharing information. Perhaps, the hypothesis concludes.

*H5: Work overload has a positive and significant impact on knowledge hiding behavior*.

#### The Mediating Role of Work Overload

In today’s business world, knowledge sharing is critical for surviving in today’s competitive environment. IC accelerates the workload, eventually making the employees incapable of fulfilling the job requirement. The focus of this investigation makes the role of workload a notable factor driving knowledge-sharing behavior. Knowledge sharing presents positive affectivity for dealing with workplace hardships (e.g., WO; Deng et al., 2018). In contrast, the internal competitive pressure lengthens the completion of the task, thereby limiting the exchange of knowledge. Given the articulation, the study shows that the competitive pressure to outperform others makes individuals exhibit KHB (Hernaus et al., 2019). Similarly, the research states that the internal competitive environment drives the employees to bear the workload, thus hiding the individual’s knowledge (Sofyan et al., 2021).

Predominately, with the increased competitiveness, WO has become a significant area that needs investigation. Job overload alludes to the situation in which employees lose work control, substantially requiring extra time and energy. This inability to cope with the increasing competitiveness (i.e., job requirement) leads the individual to experience job strain and pressure (Roja et al., 2019). Undoubtedly, WO makes individuals experience work pressure and job exhaustion. In support, the study explains that increased workplace competition leads the employees to exhibit counterproductive behavior (e.g., KHB; Shen and Kuang, 2021).

Along with this, the excessive employment workload triggers stress among the employees. In response to the increasing workload, the employees tend to act negatively toward the task, thus lowering their willingness to share the knowledge with their co-workers (Khalid et al., 2020). The psychological contract associated with heavy workload improves the degree of failure. In particular, this frustrated feeling prompts the individual to hide the information from the other fellow members. In explaining this notion, the study reveals that the escalating workload elevates the psychological factor (e.g., stress) among the individuals, subsequently bringing adverse performance outcomes (i.e., KHB; De Clercq et al., 2019). Consequently, based on the prior literature, we developed the following hypothesis.

*H5a: Work overloads mediate the relationship between interpersonal competition and knowledge hiding behavior.*

**FIGURE 1:**
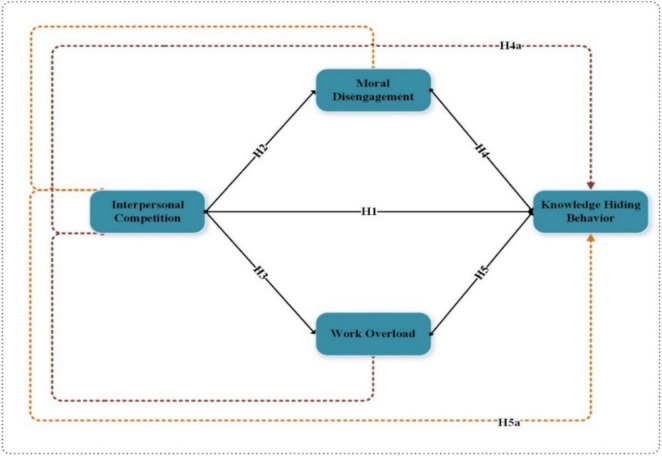
Conceptual framework.

Figure 1 shows study-dependent, -independent, mediating, and moderating variables.

## Methodology

This study’s aims to investigate the interpersonal competition effect on KHB, MD, and WO. Additionally, the study examines the mediating role of MD and WO between interpersonal competition and KHB. Therefore, this research carried out explanatory through a quantitative method to empirically investigate the variables and test hypotheses. The population of China was chosen as the target population of study and data collected by employees employed in manufacturing companies with convenient sampling techniques. The questionnaire survey was used to collect data by electronically and with a cross-sectional approach. A total of 450 questionnaires were distributed to employees, and 393 were returned. After subtracting the wrong filled questionnaires, 361 questionnaires were used for analysis with an 80% response rate.

The measurement items of interpersonal competition were adopted from the study of Lee (2020), measurement items of KHB adopted from the studies of Nguyen et al. (2022). Further, the measurement items of MD and WO were adopted from the studies Moore et al. (2012) and Karatepe (2013), respectively.

## Results

Table 1 provides the complete detail of the demographic characteristics of respondents who participated in this study.

**TABLE 1:** Demographic characteristics.

Items	Frequency (*N* = 361)	(%)
Male	170	47.1
Female	191	52.9
**Age**		
19–30	90	24.9
31–40	111	30.7
41–50	89	24.7
51–60	34	9.4
>60	37	10.2
**Edu**		
Intermediate	54	15
Bachelor	129	35.7
Master	109	30.2
MPhil/Others	69	19.1
**MS**		
Single	258	71.5
Married	103	28.5

Over 361 collected questionnaires, 191 useful responses were received from the women (52.9%) and 170 from the men (47.1%). The respondents were asked for their age. As a result, 111 (30.7%) had 31 to 40 years, 90 (24.9%) of them had 19 to 30 years, 89 (24.7%) of them had 41 to 50 years, 37 (10.2%) of them had more than 60 years, and 34 (9.4%) of the respondents had 51 to 60 years. The respondents were also to specify their educational level. As a result, 129 (35.7%) of them had bachelor, 109 (30.2%) had master, 69 (19.1%) had MPhil or other degrees, and 54 (15%) of the respondents had intermediate education. In specifying the marital status, 258 (71.5%) of the respondents were single, whereas 103 (28.5%) were married.

### Common Method Bias

This research also applied the common method bias using Harman’s single-factor approach. The variance extracted by one single factor is 13.767%, which is less than 50%, indicating no common method bias in this study (Podsakoff et al., 2003).

#### Assessment of Measurement Model

Table 2 shows that the average variance extracted (AVE), which reflects the overall variance in the indicators accounted for by the latent construct, was 0.698, 0.706, 0.654, 0.639, and 0.711 for competitive anxiety (CA), sense of rivalry (SR), MD, WO, and KHB, respectively. All values were above the cutoff 0.5 as suggested by Nunnally (1994).

**TABLE 2:** Reliability and validity analysis (zero-order).

Construct	Items	Loading	α	CR	AVE
Competitive anxiety	CA_1	0.818	0.920	0.920	0.698
	CA_2	0.803			
	CA_3	0.860			
	CA_4	0.837			
	CA_5	0.857			
Sense of rivalry	SR_1	0.841	0.905	0.905	0.706
	SR_2	0.884			
	SR_3	0.841			
	SR_4	0.792			
Moral disengagement	MD_1	0.840	0.938	0.938	0.654
	MD_2	0.863			
	MD_3	0.768			
	MD_4	0.806			
	MD_5	0.772			
	MD_6	0.778			
	MD_7	0.814			
	MD_8	0.822			
Work overload	WO_1	0.890	0.876	0.875	0.639
	WO_2	0.733			
	WO_3	0.764			
	WO_4	0.801			
Knowledge hiding behavior	KHB_1	0.812	0.967	0.967	0.711
	KHB_10	0.831			
	KHB_11	0.876			
	KHB_12	0.871			
	KHB_2	0.826			
	KHB_3	0.861			
	KHB_4	0.864			
	KHB_5	0.841			
	KHB_6	0.857			
	KHB_7	0.798			
	KHB_8	0.869			
	KHB_9	0.810			

The composite reliability (CR), which depicts the degree to which the construct indicators indicate the latent construct, was 0.920, 0.905, 0.938, 0.875, and 0.967 for CA, SR, MD, WO, and KHB, respectively. All values exceeded the recommended value of 0.6 as recommended by Bagozzi and Yi (1988).

The Cronbach’s alpha, which describes the degree to which a measure is error-free, was 0.920, 0.905, 0.938, 0.876, and 0.967 for CA, SR, MD, WO, and KHB, respectively. All values were above the threshold of 0.7, as suggested by Nunnally (1994).

As shown in Table 3, the inter-correlations between the zero-order constructs ranged between 0.295 (correlation between MD and SR) and 0.569 (correlation between KHB and WO), which were below the threshold of 0.85 (Kline, 2005).

**TABLE 3:** Discriminant validity analysis (Fornell–Larcker and HTMT, zero-order).

Constructs	1	2	3	4	5
Competitive anxiety	0.835	0.434	0.391	0.426	0.341
Knowledge hiding behavior	0.434	0.843	0.555	0.365	0.567
Moral disengagement	0.390	0.557	0.809	0.294	0.301
Sense of rivalry	0.428	0.366	0.295	0.84	0.302
Work overload	0.343	0.569	0.305	0.302	0.799

*Values on the diagonal (italicized) represent the square root of the average variance extracted while down-off diagonals are correlations and top-off diagonals are heterotrait–monotrait (HTMT) values.*

Further, as shown in Table 3, the analysis indicated that the value of the off-diagonal elements was smaller than the value of the square root of AVE on the diagonal. Therefore, it confirms that each zero-order latent construct measurement was totally discriminating to each other based on the Fornell–Larcker approach (Fornell and Larcker, 1981).

Table 3 also represents the HTMT values of zero-order constructs, which all were below the threshold of 0.90, ranging between 0.301 (HTMT between MD and WO) and 0.567 (HTMT between KHB and WO). Therefore, it confirms that each zero-order latent construct measurement was totally discriminating to each other (Henseler et al., 2009).

**FIGURE 2:**
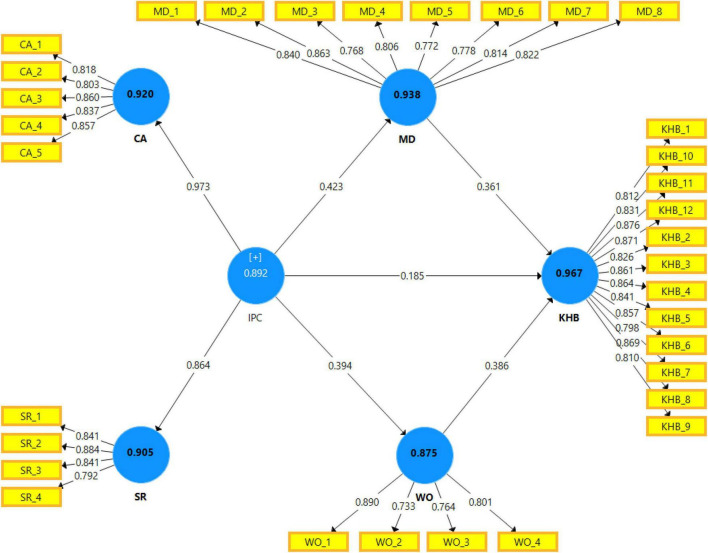
Graphical representation of assessment of measurement model (zero-order).

Figure 2 represents the assessment of the measurement model in the graph. The latent constructs were shown in circle shapes, whereas the items were shown in rectangle shapes. The values on the paths between constructs and items represent the factor loadings of those items on the construct. Also, the values on the two paths from interpersonal competition (IPC) to CA and sense of rivalry (SR) refer to the factor loading of the two zero-order constructs (i.e., CA and SR) on the second-order construct (i.e., IPC). The values inside the latent construct represent the value of Cronbach’s alpha.

Table 4 represents the reliability and convergent validity of interpersonal competition as the only second-order construct in this study with its two zero-order sub-constructs (i.e., CA and SR). The results indicated that the factor loading of CA and SR on interpersonal competition was 0.973 and 0.864, respectively, above the cutoff 0.6. The AVE was 0.847, above the cutoff 0.5. The CR was 0.917, above the cutoff 0.6 and Cronbach’s alpha was 0.833, above the cutoff 0.7 (Bagozzi and Yi, 1988; Nunnally, 1994).

**TABLE 4:** Reliability and validity analysis (second-order).

Construct	Items	Loading	α	CR	AVE
Interpersonal competition	Competitive anxiety	0.973	0.833	0.917	0.847
	Sense of rivalry	0.864			

As shown in Table 5, the inter-correlations between the hypothesized constructs ranged between 0.305 (correlation between MD and WO) and 0.608 (correlation between interpersonal competition and KHB), which were below the threshold of 0.85 as suggested by Kline (2005).

**TABLE 5:** Discriminant validity analysis (Fornell—Larcker and HTMT, second-order).

Constructs	1	2	3	4
1.Interpersonal competition	0.632	0.611	0.524	0.492
2.Knowledge hiding behavior	0.608	0.843	0.555	0.567
3.Moral disengagement	0.524	0.557	0.809	0.301
5.Work overload	0.489	0.569	0.305	0.799

*Values on the diagonal (italicized) represent the square root of the average variance extracted while down-off diagonals are correlations and top-off diagonals are HTMT values.*

Further, as shown in Table 5, the analysis indicated that the value of the off-diagonal elements was smaller than the value of the square root of AVE on the diagonal. Therefore, it confirms that each hypothesized latent construct measurement was totally discriminating to each other based on the Fornell–Larcker approach (Fornell and Larcker, 1981; Hair et al., 2014).

Table 5 also represents the HTMT values of hypothesized constructs which all were below the threshold of 0.90, ranging between 0.301 (HTMT between MD and WO) and 0.567 (HTMT between KHB and WO). Therefore, it confirms that each hypothesized latent construct measurement was totally discriminating to each other (Henseler et al., 2015).

As shown in Table 6, the variance influence factor (VIF) of interpersonal competition, MD, and WO in predicting KHB were 1.65, 1.384, and 1.320, respectively. Further, the VIF of interpersonal competition in predicting MD and WO was 1. All values were below the threshold of 3.3. Therefore, the model can be considered free of collinearity (Kock, 2015).

**TABLE 6:** Variance influence factor (second-order).

Constructs	1	2	3	4
1. Interpersonal competition		1.65	1	1
2. Knowledge hiding behavior				
3. Moral disengagement		1.384		
4. Work overload		1.32		

### Structural Model

#### Hypotheses Testing

As shown in Table 7, all hypothesized direct effect paths were statistically significant because of a *p*-value less than the standard level of 0.05. Therefore, hypotheses H1 to H5 were all supported. The following sub-sections discuss the results of path analysis concerning the above direct effect hypotheses:

**TABLE 7:** Hypotheses testing direct effect.

Hypothesis	Direct	Std.	Std.	*t*	*p*
	Relationships	*Beta*	Error	Values	Values
H1	IPC→KHB	0.282	0.123	2.297	**
H2	IPC→MD	0.524	0.066	7.887	***
H3	IPC→WO	0.489	0.075	6.489	***
H4	MD→KHB	0.306	0.091	3.38	**
H5	WO→KHB	0.338	0.091	3.727	***

Indicates significant paths: **p < 0.01, ***p < 0.001, and NS = not significant.

As shown in Table 7, the *t*-value and *p*-value of interpersonal competition in predicting the KHB were 2.297 and less than 0.01, respectively. It means that the probability of getting an at-value as large as 2.297 in absolute value is less than 1%. In other words, the regression weight for interpersonal competition in the prediction of KHB is significantly different from zero at the 0.01 level (two-tailed). Thus, H1 was supported. The standardized path coefficient was 0.282, indicating a positive relationship. When interpersonal competition goes up by one standard deviation, KHB increases by 0.282 standard deviations.

The results indicated that the probability of getting a *t*-value as large as 7.887 in absolute value is less than 0.1%. In other words, the effect of interpersonal competition on MD is positively significant at 0.001 level with the standardized path coefficient of 0.524. Therefore, hypothesis H2 was supported.

The results indicated that the probability of getting a *t*-value as large as 6.489 in absolute value is less than 0.1%. In other words, the effect of IC on WO is positively significant at 0.001 level with the standardized path coefficient of 0.489. Therefore, hypothesis H3 was supported.

The results indicated that the probability of getting a *t*-value as large as 3.380 in absolute value is less than 1%. In other words, the effect of MD on KHB is positively significant at 0.01 level with the standardized path coefficient of 0.306. Therefore, hypothesis H4 was supported.

The results indicated that the probability of getting a *t*-value as large as 3.727 in absolute value is less than 0.1%. In other words, the effect of WO on KHB is positively significant at 0.001 level with the standardized path coefficient of 0.338. Therefore, hypothesis H5 was supported.

As shown in Table 8, both hypothesized mediation effect paths were statistically significant because of a *p*-value less than the standard level of 0.05. Therefore, hypotheses H4a and H5a were both supported. The following sub-sections discuss the results of path analysis in relation to the above mediation effect hypotheses:

**TABLE 8:** Hypotheses testing mediation effect.

Hypothesis	Mediation/Indirect	Std.	Std.	*t*	*p*
	Relationships	*Beta*	Error	Values	Values
H4a	IPC→MD→KHB	0.160	0.047	3.377	**
H5a	IPC→WO→KHB	0.165	0.043	3.866	***

Indicates significant paths: **p < 0.01, ***p < 0.001, and NS = not significant.

As shown in Table 8, the result of bootstrapping indicated that the indirect effect of interpersonal competition on KHB through MD was positive and statistically significant at 0.01 level; β = 0.160, *t*-value = 3.377, *p* < 0.01. This result, along with the significant effect of interpersonal competition on KHB (from Table 7), indicated that MD partially mediates the relationship between interpersonal competition and KHB. Therefore, hypothesis H4a was supported.

As shown in Table 8, the result of bootstrapping indicated that the indirect effect of interpersonal competition on KHB through WO was positive and statistically significant at 0.001 level; β = 0.165, *t*-value = 3.866, *p* < 0.001. This result along with the significant effect of interpersonal competition on KHB (from Table 7) indicated that WO partially mediates the relationship between interpersonal competition and KHB. Therefore, hypothesis H5a was supported.

**FIGURE 3:**
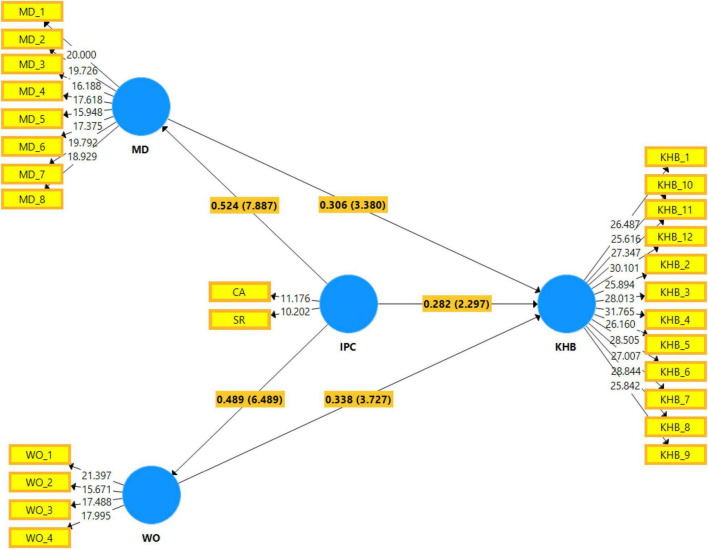
Graphical representation of the structural model.

Figure 3 represents the results of path analysis in the structural model in the graph. The values on the paths between constructs represent the standardized coefficient of the direct effects. The values on the parentheses represent *t*-values of such effect. The values inside the dependent latent construct represent not-adjusted *R*-square (*R*^2^) values.

As shown in Table 9, the adjusted value of *R*^2^, which represents the portion of the variance in the dependent variable explained by its predictors, for KHB, MD, and WO as three dependent variables in this study was 0.530, 0.272, and 0.237, respectively. This indicates, for example, that 53% of variations in KHB are explained by its three predictors (i.e., interpersonal competition, MD, and WO). Overall findings showed that the *R*^2^ values satisfy the requirement for the 0.30 cutoff value.

**TABLE 9:** Quality criteria values.

Latent variables	*R* ^2^	*R* ^2*Adj*^	*Q* ^2^	*F* ^2^
KHB	0.534	0.530	0.339	
MD	0.274	0.272	0.103	
WO	0.239	0.237	0.087	
IPC→KHB (H1 – small)				0.104
IPC→MD (H2 – large)				0.378
IPC→WO (H3 – medium)				0.314
MD→KHB (H4 – small)				0.145
WO→KHB (H5 – medium)				0.185

The value of *Q*^2^, which represents cross-validated redundancy, for KHB, MD, and WO was 0.339, 0.103, and 0.087, respectively, far greater than zero, which refers to predictive relevance of the model as suggested by Chin (2010). In sum, the model exhibits acceptable fit and high predictive relevance.

The value of *F*-squared *F*^2^, which represents the size of an effect by considering the changes in *R*^2^, was different for different paths. According to Carte and Russell (2003), there is no effect size for *F*^2^ below 0.02, small if the *F*^2^ ranges within 0.02 to 0.15, medium if ranges within 0.15 to 0.35, and large for the *F*^2^ above 0.35. Therefore, the effect sizes of interpersonal competition and MD on KHB were small (i.e., *F*^2^ = 0.104 and 0.145 for H1 and H4, respectively). The effect sizes of interpersonal competition on MD (i.e., *F*^2^ = 0.314 for H3) and the effect sizes of WO on KHB (i.e., *F*^2^ = 0.185 for H5) were medium. The effect size of interpersonal competition on MD was large; *F*^2^ = 0.378 for H5.

**FIGURE 4:**
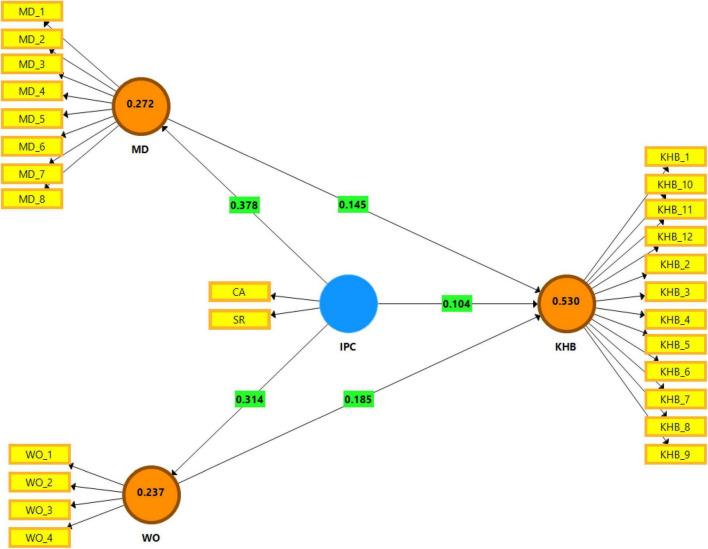
Graphical representation of *R*^2^ and *F*^2^.

Figure 4 represents the results of path analysis in the structural model in the graph. The values on the paths between constructs represent the *F*^2^ or effect size of the path. The values inside the dependent latent construct represent adjusted *R*^2^ values.

## Discussion

In today’s business environment, the growing interpersonal competition has significantly recorded unfavorable results (e.g., KHB, MD, and WO). Section “Discussion” presents the study outcomes in the light of previous empirical reviews. The current research highlights the direct and indirect relationships among KHB, MD, and WO. In particular, in this study, the variables such as KHB and WO had treated as the mediators. However, the research findings show that all the hypotheses were accepted and approved.

Arguably, interpersonal competition widely observed in today’s work setting has accelerated workplace KHB. The highly competitive internal environment affects organizations’ functions, thus adversely affecting employee knowledge sharing behavior. Given the illustration, the study shows that knowledge hiding in the competitive business environment produces undesirable organizational outcomes such as counterproductive work behavior (Hernaus et al., 2019). Similarly, the studies reveal firm’s internal competition influences unethical behavior (Li et al., 2018) and WO (Qasim et al., 2020). Perhaps, our study findings were found significantly consistent with prior research, thus making us to accept H1, H2, and H3.

Furthermore, MD concerning KHB has gained considerable management attention in the last few years. In particular, this growing construct (i.e., MD) motivates the individual to demonstrate unethical behavior. Accordingly, the previous verifications indicate that MD leads to KHB (Arain et al., 2020). Perhaps, our study results had recorded similar results, thus concluding a positive and significant relationship between MD and KHB.

Knowledge serves as a strategic tool, enhancing the firm’s competitiveness. The previous studies investigated the influence of interpersonal competition concerning MD and WO. Accordingly, the prior research reveals that MD promotes KHB at the workplace (Peng et al., 2021), thus making employees achieve internal competitiveness. Similarly, this article also explores the mediating effect of the WO nexus on interpersonal relationships and KHB. The findings were consistent with the prior literature that indicates that excessive workload makes the individual hide the company’s knowledge, thus winning the internal competition (Arain et al., 2020). Hence, based on these findings, all the hypotheses had significantly supported and accepted (e.g., H4, H4a, H5, and H5a).

## Conclusion and Implications

The importance of knowledge managing and sharing in an organization leads to successful change and innovation. Despite various efforts to promote knowledge sharing at the workplace, workers are not ready to share the knowledge because of some constraints that lead to KHB. In the current era of technological advancement, organizations have become highly dependent on knowledge assets for their survival. Knowledge is the most fundamental organizational resource that influences the employees’ behavior. This study highlights the role of KHB in the internal competitive environment. The study objective was to explore the different factors that lead to KHB among employees. This study concluded that interpersonal competition positively and significantly affects KHB, MD, and WO. Moreover, the study established that MD and WO positively and significantly impact KHB. According to the results of this study, MD WO significantly mediates interpersonal competition and KHB.

This study offers a theoretical foundation for recognizing and demonstrating the current and possible innumerable knowledge hiding activities by workers in organizations. Therefore, many studies have been done on knowledge sharing, but few research found on KHB, and this unfavorable behavior requires more investigation. This evidence from this study gives new context and recommendations to managers that KHB is challenging as present and future events have a negative impact on individuals and organizations.

This study delivers an understanding of knowledge hiding from the last 10 years. Moreover, managers are investing and facilitating in the sharing of knowledge. Still, the KHB found commonly in an organization affects the innovation and change in the organization and harms interpersonal interactions. This study provides new insights on how supervisors can prevent knowledge hiding events and how to provide ease to share effective information among individuals in organizations. Knowledge sharing is the most significant element of human resource management, and administrators play an effective role in knowledge sharing among workers to increase individual, organizational, and team performance. This research suggests that human resource management should adopt and implement effective strategies to facilitate the employees in knowledge sharing to eliminate the KHB. Moreover, this research discovered that managers need to focus on the negative transformation, influential and perceived disengagement in proposed events. This study has theoretical contributes to research objectives and concludes that managers should understand and focus on present and future events that causes to increase the KHB and how managers adopt the strategies to reduce this behavior in the organization.

### Limitations and Future Directions

The study proposes that more research should focus on important factors that lead to KHB and empirically test whether those factors have negative or positive outcomes. At the same time, the study is limited just to three factors: interpersonal competition, MD, and WO. The study investigation is limited to KHB among employees. Still, some more research should be conducted with human resource management that they are implementing effective strategies to reduce the knowledge hiding in the organization. This study fails to provide broader knowledge on the literature of KHB; more research should be focused on the broader concept between the organization and members with bibliometric analysis to accomplish the balanced and more comprehensive undersetting of KHB.

## Data Availability Statement

The raw data supporting the conclusions of this article will be made available by the author, without undue reservation.

## Ethics Statement

Ethical review and approval was not required for the study on human participants in accordance with the local legislation and institutional requirements. The patients/participants provided their written informed consent to participate in this study.

## Author Contributions

The author solely contributed to this study.

## Conflict of Interest

The author declares that the research was conducted in the absence of any commercial or financial relationships that could be construed as a potential conflict of interest.

## Publisher’s Note

All claims expressed in this article are solely those of the authors and do not necessarily represent those of their affiliated organizations, or those of the publisher, the editors and the reviewers. Any product that may be evaluated in this article, or claim that may be made by its manufacturer, is not guaranteed or endorsed by the publisher.

## References

[B1] Abdullah M. I. Huang D. Sarfraz M. Ivascu L. Riaz A. (2021). Effects of internal service quality on nurses’ job satisfaction, commitment and performance: mediating role of employee well-being. *Nurs. Open* 8 607–619. 10.1002/nop2.665 33570299 PMC7877139

[B2] Abdullah M. I. Sarfraz M. Arif A. Azam A. (2018a). An extension of the theory of planned behavior towards brand equity and premium price. *Pol. J. Manag. Stud.* 18 20–32. 10.17512/pjms.2018.18.1.02

[B3] Abdullah M. I. Sarfraz M. Qun W. Javaid N. (2018b). Drivers of green supply chain management. *LogForum* 14 437–447. 10.1007/s11356-021-16638-9 34617237

[B4] Ajaz A. Shenbei Z. Sarfraz M. (2020). Delineating the influence of boardroom gender diversity on corporate social responsibility, financial performance, and reputation. *LogForum* 16 61–74.

[B5] Anand A. Centobelli P. Cerchione R. (2020). Why should I share knowledge with others? A review-based framework on events leading to knowledge hiding. *J. Organ. Change. Manag.* 33 379–399. 10.1108/JOCM-06-2019-0174

[B6] Anand P. Hassan Y. (2019). Knowledge hiding in organizations: everything that managers need to know. *Dev. Lear. Organ. Int. J.* 33 12–15. 10.1108/DLO-12-2018-0158

[B7] Arain G. A. Bhatti Z. A. Hameed I. Fang Y.-H. (2020). Top-down knowledge hiding and innovative work behavior (IWB): a three-way moderated-mediation analysis of self-efficacy and local/foreign status. *J. Knowl. Manag.* 24 127–149. 10.1108/JKM-11-2018-0687

[B8] Babalola M. T. Ren S. Ogbonnaya C. Riisla K. Soetan G. T. Gok K. (2022). Thriving at work but insomniac at home: understanding the relationship between supervisor bottom-line mentality and employee functioning. *Hum. Relations* 75 33–57. 10.1177/0018726720978687

[B9] Bagozzi R. P. Yi Y. (1988). On the evaluation of structural equation models. *J. Acad. Mark. Sci.* 16 74–94.

[B10] Bandura A. Barbaranelli C. Caprara G. V. Pastorelli C. (1996). Mechanisms of moral disengagement in the exercise of moral agency. *J. Pers. Soc. Psychol.* 71 364–374. 10.1037/0022-3514.71.2.364

[B11] Bavik A. (2020). A systematic review of the servant leadership literature in management and hospitality. *Int. J. Contemp. Hosp. Manag.* 32 347–382. 10.1108/IJCHM-10-2018-0788

[B12] Boz Semerci A. (2019). Examination of knowledge hiding with conflict, competition and personal values. *Int. J. Confl. Manag.* 30 111–131. 10.1108/IJCMA-03-2018-0044

[B13] Bunjak A. Èerne M. Nagy N. Bruch H. (2021). Job demands and burnout: the multilevel boundary conditions of collective trust and competitive pressure. *Hum. Relations* 001872672110598. 10.1177/00187267211059826

[B14] Burmeister A. Fasbender U. Deller J. (2018). Being perceived as a knowledge sender or knowledge receiver: a multistudy investigation of the effect of age on knowledge transfer. *J. Occup. Organ. Psychol.* 91 518–545. 10.1111/joop.12208

[B15] Carte T. A. Russell C. J. (2003). In pursuit of moderation: nine common errors and their solutions. *MIS Q.* 27 479–501. 10.2307/30036541

[B16] Chin W. W. (2010). “How to write up and report PLS analyses,” in *Handbook of Partial Least Squares. Springer Handbooks of Computational Statistics*, eds Esposito Vinzi V. Chin W. Henseler J. Wang H. (Berlin: Springer), 655–690. 10.3390/foods10092224

[B17] Connelly C. E. Černe M. Dysvik A. Škerlavaj M. (2019). Understanding knowledge hiding in organizations. *J. Organ. Behav.* 40 779–782. 10.1002/job.2407

[B18] Converso D. Loera B. Molinengo G. Viotti S. Guidetti G. (2018). Not all academics are alike: first validation of the academics’ quality of life at work scale (AQoLW). *Front. Psychol.* 9:2408. 10.3389/fpsyg.2018.02408 30559699 PMC6286960

[B19] De Clercq D. Haq I. U. Azeem M. U. (2019). Time-related work stress and counterproductive work behavior. *Pers. Rev.* 48 1756–1781. 10.1108/PR-07-2018-0241

[B20] Deng H. Coyle-Shapiro J. Yang Q. (2018). Beyond reciprocity: a conservation of resources view on the effects of psychological contract violation on third parties. *J. Appl. Psychol.* 103 561–577. 10.1037/apl0000272 29265825

[B21] Engelbrecht G. J. Beer L. T. Schaufeli W. B. (2020). The relationships between work intensity, workaholism, burnout, and self-reported musculoskeletal complaints. *Hum. Factors Ergon. Manuf. Serv. Ind.* 30 59–70. 10.1002/hfm.20821

[B22] Feng J. Wang C. (2019). oes abusive supervision always promote employees to hide knowledge? From both reactance and COR perspectives. *J. Knowl. Manag.* 23 1455–1474. 10.1108/JKM-12-2018-0737

[B23] Fornell C. Larcker D. F. (1981). *Structural Equation Models with Unobservable Variables and Measurement Error: Algebra and Statistics.* Los Angeles, CA: Sage Publications Sage CA.

[B24] Han M. S. Masood K. Cudjoe D. Wang Y. (2020). Knowledge hiding as the dark side of competitive psychological climate. *Leadersh. Organ. Dev. J*. 42 195–207. 10.1108/lodj-03-2020-0090

[B25] Hair J. F. Jr. Sarstedt M. Hopkins L. Kuppelwieser V. G. (2014). Partial least squares structural equation modeling (PLS-SEM): an emerging tool in business research. *Eur. Bus. Rev.* 26, 106–121. 10.1108/EBR-10-2013-0128

[B26] Harris L. C. He H. (2019). Retail employee pilferage: a study of moral disengagement. *J. Bus. Res.* 99 57–68. 10.1016/j.jbusres.2019.02.008

[B27] Henseler J. Ringle C. M. Sarstedt M. (2015). A new criterion for assessing discriminant validity in variance-based structuralequation modeling. *J. Acad. Mark. Sci.* 43, 115–135.

[B28] Henseler J. Ringle C. M. Sinkovics R. R. (2009). “The use of partial least squares path modeling in international marketing,” in *New Challenges to International Marketing*, eds Sinkovics R. R. Ghauri P. N. (Bingley: Emerald Group Publishing Limited). 10.2196/jmir.3122

[B29] Hernaus T. Cerne M. Connelly C. Poloski Vokic N. Škerlavaj M. (2019). Evasive knowledge hiding in academia: when competitive individuals are asked to collaborate. *J. Knowl. Manag.* 23 597–618. 10.1108/JKM-11-2017-0531

[B30] Hobfoll S. E. Halbesleben J. Neveu J.-P. Westman M. (2018). Conservation of resources in the organizational context: the reality of resources and their consequences. *Ann. Rev. Organ. Psychol. Organ. Behav.* 5 103–128. 10.1038/s41598-020-71501-0 32943683 PMC7498602

[B31] Jabeen N. Anwar-ul-Haq M. (2021). Understanding narcissists’ knowledge hiding behavior: a moral disengagement mechanism perspective. *Rev. Manag. Sci.* 3 24–41. 10.53909/admin.v3i2.93

[B32] Jahanzeb S. De Clercq D. Fatima T. (2020). Bridging the breach: using positive affectivity to overcome knowledge hiding after contract breaches. *J. Psychol.* 154 249–272. 10.1080/00223980.2019.1705235 31916918

[B33] Jha J. K. Pandey J. Varkkey B. (2019). xamining the role of perceived investment in employees’ development on work-engagement of liquid knowledge workers. *J. Glob. Oper. Strateg. Sourcing* 12 225–245. 10.1108/JGOSS-08-2017-0026

[B34] Jiang Z. Hu X. Wang Z. Jiang X. (2019). Knowledge hiding as a barrier to thriving: the mediating role of psychological safety and moderating role of organizational cynicism. *J. Organ. Behav.* 40 800–818. 10.1002/job.2358

[B35] Kalra A. Agnihotri R. Talwar S. Rostami A. Dwivedi P. K. (2021). Effect of internal competitive work environment on working smart and emotional exhaustion: the moderating role of time management. *J. Bus. Ind. Mark.* 36 269–280. 10.1108/JBIM-02-2019-0094

[B36] Karatepe O. M. (2013). The effects of work overload and work-family conflict on job embeddedness and job performance: The mediation of emotional exhaustion. *Int. J. Contemp. Hosp. Manag*. 25 614–634. 10.1108/09596111311322952

[B37] Khalid M. Bashir S. Khan A. K. Abbas N. (2018). When and how abusive supervision leads to knowledge hiding behaviors. *Leadersh. Organ. Dev. J.* 39 794–806. 10.1108/LODJ-05-2017-0140

[B38] Khalid M. Gulzar A. Karim Khan A. (2020). When and how the psychologically entitled employees hide more knowledge? *Int. J. Hosp. Manag.* 89:102413. 10.1016/j.ijhm.2019.102413

[B39] Khan S. Liang D. Anjum M. A. Shah S. J. (2021). Linking perceived market competition threat to moral disengagement: the roles of fear of failure and moral relativism. *Curr. Psychol.* 40 4086–4100. 10.1007/s12144-019-00365-z

[B40] Khan S. Liang D. Shah A. M. Ullah R. (2018). “The buffering role of ethical leadership in moral disengagement: anticompetitive behavioral tendency link,” in *Proceedings of the European Conference on Management, Leadership & Governance.* Utrecht.

[B41] Kline T. (2005). *Psychological Testing: A Practical approach to Design and Evaluation.* Thousand Oaks, CA: Sage.

[B42] Kock N. (2015). Common method bias in PLS-SEM: a full collinearity assessment approach. *Int. J. Collabor.* 11, 1–10.

[B43] Kumar Jha J. Varkkey B. (2018). Are you a cistern or a channel? Exploring factors triggering knowledge-hiding behavior at the workplace: evidence from the Indian R&D professionals. *J. Knowl. Manag.* 22 824–849. 10.1108/JKM-02-2017-0048

[B44] Lanke P. (2018). Knowledge hiding: impact of interpersonal behavior and expertise. *Hum. Resour. Manag. Int. Dig.* 26 30–32. 10.1108/HRMID-01-2018-0010

[B45] Lee H.-W. (2020). Interpersonal competition in organization: an investigation of antecedents. *Int. J. Manpow*. 41 1363–1383. 10.1080/00223980.2019.1578191 30924730

[B46] Li Y. Feng T. Jiang W. (2018). How competitive orientation influences unethical decision-making in clinical practices? *Asian Nurs. Res.* 12 182–189. 10.1016/j.anr.2018.07.001 30056142

[B47] Liu X. Kim T.-Y. (2020). Team Competitive climate, creative personality, and unethical behaviors. *Acad. Manag. Proc.* 2020:16460. 10.5465/AMBPP.2020.16460abstract

[B48] Moore C. Detert J. R. Klebe Treviño L. Baker V. L. Mayer D. M. (2012). Why employees do bad things: moral disengagement and unethical organizational behavior. *Pers. Psychol.* 65 1–48. 10.1111/j.1744-6570.2011.01237.x

[B49] Moore C. Mayer D. M. Chiang F. F. T. Crossley C. Karlesky M. J. Birtch T. A. (2019). Leaders matter morally: the role of ethical leadership in shaping employee moral cognition and misconduct. *J. Appl. Psychol.* 104 123–145. 10.1037/apl0000341 30221953

[B50] Nguyen T.-M. Malik A. Budhwar P. (2022). Knowledge hiding in organizational crisis: the moderating role of leadership. *J. Bus. Res.* 139 161–172. 10.1016/j.jbusres.2021.09.026 34667337 PMC8516615

[B51] Nunnally J. C. (1994). *Psychometric Theory 3E.* New York, NY: Tata McGraw-hill education.

[B52] Peng H. Bell C. Li Y. (2021). How and when intragroup relationship conflict leads to knowledge hiding: the roles of envy and trait competitiveness. *Int. J. Confl. Manag.* 32 383–406. 10.1108/IJCMA-03-2020-0041

[B53] Pérez-Salazar M. del R. Aguilar-Lasserre A. A. Cedillo-Campos M. G. Juárez-Martínez U. Posada-Gómez R. (2019). Processes and measurement of knowledge management in supply chains: an integrative systematic literature review. *Int. J. Prod. Res.* 57 2136–2159. 10.1080/00207543.2018.1521530

[B54] Podsakoff P. M. MacKenzie S. B. Lee J.-Y. Podsakoff N. P. (2003). Common method biases in behavioral research: a critical review of the literature and recommended remedies. *J. Appl. Psychol.* 88:879. 10.1037/0021-9010.88.5.879 14516251

[B55] Qasim S. Bahadar S. Muhammad W. Hazrat B. (2020). An empirical analysis of work overload, organizational commitment and turnover intentions among employees of banking sector. *J. Bus. Soc. Rev. Emerg. Econ.* 6 781–788. 10.26710/jbsee.v6i2.1225

[B56] Qin X. Dust S. B. DiRenzo M. S. Wang S. (2020). Negative creativity in leader-follower relations: a daily investigation of leaders’ creative mindset, moral disengagement, and abusive supervision. *J. Bus. Psychol.* 35 665–682. 10.1007/s10869-019-09646-7

[B57] Rani H. Arain G. A. Kumar A. Shaikh I. R. (2018). Interplay between trust and distrust in the workplace: examining the effect of psychological contract breach on organizational disidentification. *J. Asia Bus. Stud.* 12 1–16. 10.1108/JABS-02-2015-0022

[B58] Raza S. A. Najmi A. Shah N. (2018). Transferring knowledge from universities to organizations by business students. *J. Workplace Learn.* 30 199–215. 10.1108/JWL-06-2016-0054

[B59] Roja Z. Kalkis H. Tetere I. Roja I. (2019). “Stress at work and physical load in professional sport,” in *Proceedings of the AHFE International Conference on Physical Ergonomics and Human Factors, 2018*, Orlando, FL, 335–342. 10.1007/978-3-319-94484-5_35

[B60] Sahi Q. B. Ahmad M. (2019). Impact of Job insecurity and moral disengagement on counterproductive work behavior. *City Univ. Res. J.* 9 279–294. 10.3390/ijerph18168354 34444104 PMC8394277

[B61] Sarfraz M. Qun W. Shah S. G. M. Fareed Z. (2019). Do hierarchical jumps in CEO succession invigorate innovation? Evidence from Chinese economy. *Sustainability (Switzerland)* 11:2017. 10.3390/su11072017

[B62] Scheiner C. W. Baccrella C. V. Bessant J. Voigt K. (2018). Participation motives, moral disengagement, and unethical behaviour in idea competitions. *Int. J. Innov. Manag.* 22:1850043. 10.1142/S1363919618500433

[B63] Shah S. G. M. Sarfraz M. Fareed Z. Rehman M. A. ur, Maqbool A. Qureshi M. A. A. (2019). Whether CEO Succession via hierarchical jumps is detrimental or blessing in disguise? Evidence from Chinese listed firms. *Zagreb Int. Rev. Econ. Bus.* 22 23–41. 10.2478/zireb-2019-0018

[B64] Shehzad K. Xiaoxing L. Sarfraz M. Zulfiqar M. (2020). Signifying the imperative nexus between climate change and information and communication technology development: a case from Pakistan. *Environ. Sci. Poll. Res.* 27 30502–30517. 10.1007/s11356-020-09128-x 32468367

[B65] Shen B. Kuang Y. (2021). Assessing the relationship between technostress and knowledge hiding—a moderated mediation model. *Data Inf. Manag.* 1, 1–12. 10.2478/dim-2021-0015

[B66] Shrivastava S. Pazzaglia F. Sonpar K. (2021). he role of nature of knowledge and knowledge creating processes in knowledge hiding: reframing knowledge hiding. *J. Bus. Res.* 136 644–651. 10.1016/j.jbusres.2021.08.019

[B67] Singh S. K. (2019). Territoriality, task performance, and workplace deviance: empirical evidence on role of knowledge hiding. *J. Bus. Res.* 97 10–19. 10.1016/j.jbusres.2018.12.034

[B68] Sofyan Y. De Clercq D. Shang Y. (2021). Detrimental effects of work overload on knowledge hiding in competitive organisational climates. *Asia Pac. J. Hum. Resour.* 1, 1–31. 10.1111/1744-7941.12317

[B69] Spector P. E. Jex S. M. (1998). Development of four self-report measures of job stressors and strain: interpersonal conflict at work scale, organizational constraints scale, quantitative workload inventory, and physical symptoms inventory. *J. Occup. Health Psychol.* 3 356–367. 10.1037/1076-8998.3.4.356 9805281

[B70] Swab R. G. Johnson P. D. (2019). Steel sharpens steel: a review of multilevel competition and competitiveness in organizations. *J. Organ. Behav.* 40 147–165. 10.1002/job.2340

[B71] van de pol P. K. C. Kavussanu M. Claessens B. (2020). Moral functioning across training and competition in sport. *Int. J. Sport Exerc. Psychol.* 18 239–255. 10.1080/1612197X.2018.1511623

[B72] Wallace J. E. Buchanan T. (2019). Status differences in interpersonal strain and job resources at work. *Int. J. Confl. Manag.* 31 287–308. 10.1108/IJCMA-08-2019-0135

[B73] Wen J. Ma R. (2021). Antecedents of knowledge hiding and their impact on organizational performance. *Front. Psychol.* 12:796976. 10.3389/fpsyg.2021.796976 34987455 PMC8722472

[B74] Xiao M. Cooke F. L. (2019). Why and when knowledge hiding in the workplace is harmful: a review of the literature and directions for future research in the Chinese context. *Asia Pac. J. Hum. Resour.* 57 470–502. 10.1111/1744-7941.12198

[B75] Xiong C. Chang V. Scuotto V. Shi Y. Paoloni N. (2021). The social-psychological approach in understanding knowledge hiding within international R&D teams: an inductive analysis. *J. Bus. Res.* 128 799–811. 10.1016/j.jbusres.2019.04.009

[B76] Zhao H. Xia Q. (2019). Nurses’ negative affective states, moral disengagement, and knowledge hiding: the moderating role of ethical leadership. *J. Nurs. Manag.* 27 357–370. 10.1111/jonm.12675 30288835

